# Extramammary Paget’s Disease of the Vulva and Concomitant Premalignant/Malignant Vulvar Lesions: A Potential Challenge in Diagnosis and Treatment

**DOI:** 10.3390/curroncol30010073

**Published:** 2023-01-10

**Authors:** Nicolò Clemente, Andrea Ciavattini, Gaetano Valenti, Federica Zannier, Jacopo Di Giuseppe, Giovanni Delli Carpini, Mariasole Fichera, Anna Del Fabro, Giorgio Giorda, Gaia Goteri, Vincenzo Canzonieri, Francesco Sopracordevole

**Affiliations:** 1 Gynecological Oncology Unit, Centro di Riferimento Oncologico—National Cancer Institute, IRCCS, 33081 Aviano, Italy; 2 Gynecologic Section, Department of Odontostomatologic and Specialized Clinical Sciences, Università Politecnica delle Marche, 60123 Ancona, Italy; 3 Unit of Gynecology and Obstetrics, Department of Women’s and Children’s Health, Umberto I Hospital, 94100 Enna, Italy; 4 Pathology Unit, Centro di Riferimento Oncologico—National Cancer Institute, IRCCS, 33081 Aviano, Italy; 5 Pathology Unit, Santa Maria della Misericordia University Hospital, 33100 Udine, Italy; 6 Division of Pathological Anatomy, Department of Biomedical Sciences and Public Health, Università Politecnica delle Marche, 60123 Ancona, Italy; 7 Department of Medical, Surgical, and Health Sciences, University of Trieste, 34127 Trieste, Italy

**Keywords:** extramammary Paget’s disease, vulvar lesion, VIN, vulvar intraepithelial neoplasia, vulvar cancer, vulvar precancer, lichen sclerosus

## Abstract

**Simple Summary:**

Extramammary Paget’s disease of the vulva (EMPDV) is a rare disease. However, an association between EMPDV and other premalignant/malignant vulvar lesions is not uncommon. Due to its potential clinical implication, such an association should be carefully evaluated in all women diagnosed with and treated for EMPDV.

**Abstract:**

The aim of the present study was to evaluate the incidence of concomitant vulvar cancers or premalignant lesions in women surgically treated for extramammary Paget’s disease of the vulva (EMPDV) through a multicenter case series. The medical records of all women diagnosed with and treated for EMPDV from January 2010 to December 2020 were retrospectively analyzed. Women with EMPDV and synchronous vulvar cancer, vulvar intraepithelial neoplasia (VIN) and/or lichen sclerosus (LS) at the histology report were included in the study. A total of 69 women eligible for the present study were considered. Concomitant vulvar lesions occurred in 22 cases (31.9%). A total of 11 cases of synchronous VIN (50%) and 14 cases (63.6%) of concomitant LS were observed. One patient (4.5%) had synchronous vulvar SCC (FIGO stage 1B). Women with EMPDV and concomitant premalignant/malignant vulvar lesions had a significantly higher rate of invasive EMPDV and wider lesions with an extravulvar involvement. The specific meaning of the association between EMPDV, VIN, SCC and LS remains unclear. The potential overlapping features between different vulvar lesions highlight the importance of dedicated gynecologists and pathologists in referral centers.

## 1. Introduction

Extramammary Paget’s disease (EMPD) is an unusual skin neoplasm with an unclear pathogenesis [1]. EMPD most commonly arises as an intraepithelial neoplasm of the epidermis (primary EMPD) with Paget cells likely originating from intraepidermal portions of apocrine glands or primitive basal cells [2]. Less frequently, EMPD may occur from the epidermotropic spread of malignant cells or a direct extension from an underlying neoplasm such as gastrointestinal or genitor-urinary adenocarcinomas (secondary EMPD) [3]. 

The vulva is the most common site of involvement in patients with EMPD, accounting for 20% of all EMPD cases; however, in many patients, a perineal and/or perianal involvement (as a unique site or in association with vulvar lesions) is documented [4]. However, vulvar EMPD (EMPDV) is a rare condition, representing only 1–2% of all vulvar malignancies [5], and shows several distinctive features. Although EMPDV is usual considered to be an intraepithelial lesion, a stromal invasion is described in 13 to 40% of cases, especially in recurrent disease [1,6]. Thus, even considering the potential risk of invasion, a surgical excision remains the treatment of choice [7]. A high rate of local recurrence is another peculiarity, ranging between 20 and 70% [1,6]. According to the current literature, the influence of the surgical margin status on recurrence rates shows contrasting results [8]. This may be due to the multifocal nature of the disease and the typical skip lesion pattern of Paget cells in the epithelium as microscopic diseases often extend beyond a clinically visible lesion [9].

Exiguous data about the association between EMPDV and other vulvar lesions are available in the literature. Several cases of concurrent vulvar adenocarcinomas in patients with EMPDV have been described [10] whereas only a few cases of concomitant vulvar squamous cell carcinoma (SCC) and vulvar intraepithelial neoplasia (VIN) have been reported [11,12,13]. Thus, the potential clinical implications of this association are unknown.

Furthermore, due to a few overlapping aspects of EMPDV with HPV-induced cellular modifications, several studies have evaluated the presence of HPV in women with EMPDV [14,15]. There are also occasional reports about an association with lichen sclerosus (LS) [16], whose role as a potential risk factor for vulvar SCC has been already described [17,18] with genital warts [19] and melanomas [20].

The coexistence of EMPDV and vulvar cancers and precancers could have important implications concerning the diagnosis, therapy and proper follow-up strategies.

The aim of the present study was to evaluate the incidence of concomitant premalignant/malignant vulvar lesions in women surgically treated for EMPDV through a multicenter case series.

## 2. Materials and Methods

The medical records of all women with a first diagnosis of EMPDV who underwent a surgical excision from January 2010 to December 2020 at the Gynecological Oncology Unit, IRCCS CRO (Centro di Riferimento Oncologico) National Cancer Institute (Aviano, Italy) and the Gynecologic Section, Department of Odontostomatologic and Specialized Clinical Sciences, Università Politecnica delle Marche (Ancona, Italy) were retrospectively analyzed. Women with a biopsy diagnosis of EMPDV who underwent different treatments rather than a surgical excision (e.g., radiotherapy) were not included in the present analysis.

The pertinent clinical, surgical and histopathological data of women eligible for the present study were collected through a medical chart review. Patients with incomplete histopathological and/or clinical data were not considered for the present analysis.

Women with evidence of EMPDV and synchronous lesions (vulvar cancer, VIN and/or LS) at the histology report were included in the study. Only women diagnosed with and treated for EMPDV for the first time during the study period were considered; women with an EMPDV recurrence were excluded.

Subsequently, the main clinical characteristics of women with EMPDV and concomitant vulvar premalignant/malignant lesions were compared with those of women diagnosed only with EMPDV in the same period.

In particular, data about the age, size and location of clinically visible vulvar lesions, the rate of invasive EMPDV and a history of previous vulvar cancer were collected and compared.

VIN was classified according to the 2015 ISSVD terminology [21] in vulvar HSIL (high-grade squamous intraepithelial lesion; HPV-related and formerly known as usual-type VIN) and d-VIN (differentiated, not HPV-related). Cases diagnosed before the introduction of the 2015 ISSVD terminology were revised accordingly.

As per the routine in our institutions, all the women were asked to sign a proper informed consent before the surgery for the clinical data collection. Ethics approval for the review of the case records was obtained from the Clinical Research Ethics Committee of our institutions (CRO IRB 2014–30).

## 3. Results

From January 2010 to December 2020, 83 consecutive cases of EMPDV were diagnosed for the first time at our institutions and underwent surgical treatment. Among them, 14 patients were excluded because of incomplete clinical data available; thus, 69 women were eligible for the present study. Concomitant vulvar lesions occurred in 22 cases (31.9%). The mean age at diagnosis in the whole study cohort was 65.6 years old (SD ± 12.9).

The main characteristics of the patients with EMPDV and concomitant premalignant/malignant vulvar lesions are reported in Table 1.

The mean age at diagnosis in these women was 64.2 years old (SD ± 13). The lesion was confined to the vulva in 12 cases (54.5%) and the remaining 10 patients had lesions also involving the perineum and/or perianal region. Seven cases of multifocal lesions were found (31.8%). In this cohort, we found 7 cases (31.8%) of invasive EMPDV (type 1b) and all of them were microinvasive cancer (depth of stromal invasion < 1 mm). No case of EMPDV with an underlying adenocarcinoma of the skin (type 1c) was found.

A total of 11 cases of concomitant VIN (50%) were observed (9 cases of d-VIN and 2 of vulvar HSIL). LS was observed in 14 cases (63.6%), with coexisting VIN in 4 of them. One case (4.5%) of concomitant vulvar SCC (FIGO stage 1B) was found.

A comparison between the 22 women with EMPDV and concomitant vulvar premalignant/malignant lesions and the remaining patients diagnosed with and treated for EMPDV during the study period is shown in Table 2. Women with EMPDV and concomitant premalignant/malignant vulvar lesions showed a significantly higher rate of invasive EMPDV and wider lesions with a more frequent extravulvar involvement (perineum/perianal region). No difference in the mean age between the two groups emerged.

## 4. Discussion

Even if the vulva is the most common site of EMPD, EMPDV remains a rare clinical condition and, therefore, its true incidence and prevalence is still unknown [1]. According to the current literature, the association between EMPDV and other vulvar premalignant/malignant lesions seems to be even rarer.

To date, only 9 reports of EMPDV and concomitant vulvar premalignant/malignant lesions have been published (Table 3), with a total of 11 cases described worldwide [12,13,16,22,23,24,25,26,27].

Due to the small number of cases of concomitant EMPDV and other premalignant/malignant vulvar lesions reported, accurate data about the true incidence and prevalence of this condition are not available.

In our opinion, the coexistence of EMPDV and other vulvar premalignant/malignant lesions is not as rare as previously theorized. In our 10 years of experience, we found 22 cases of EMPDV with a concomitant VIN, LS or SCC (about one-third of all the women diagnosed with and treated for EMPDV in our institutions). To our knowledge, this is the largest series ever published, with the highest rate of concomitant EMPDV and other premalignant/malignant vulvar lesions ever reported.

The rate of coexistence of EMPDV and other premalignant/malignant vulvar lesions in our series is remarkable and, in our opinion, the small number of cases previously described in the literature probably reflects a misdiagnosis of this condition.

The specific meaning of the association between EMPDV, VIN and LS remains unclear, but it is interesting from both a histopathological and a clinical point of view.

Several authors considered the concomitant presence of EMPDV and VIN a mere coincidence [16], but, in our opinion, and considering the high rate of coexisting lesions reported in our analysis, such an association is unlikely to be merely a coincidence.

There is evidence to suggest a possibility that the association between EMPDV and VIN could be the result of a common origin from multipotent basal cells, as reported for mixed carcinomas in situ of other cutaneous sites [28]. Immunohistochemical findings seem to support this hypothesis [29]. Paget cells usually express PAS, EMA, CEA and cytokeratin CK8 and CK7, but do not express the markers of squamous cell differentiation (p53 and p40) and melanocyte markers (S100, Mart1 and HMB45). Notwithstanding, several authors found a strong expression of cytokeratin CK7 and CK8 in a few “mixed” lesions, both in Paget cells and in the areas of full-thickness squamous dysplasia, suggesting a common cell origin of these lesions [13,29,29,30].

However, regardless the etiopathogenic meaning of the association between EMPDV and other vulvar lesions, it could be important from a clinical point of view.

The vulva is a peculiar anatomical district where several premalignant and malignant lesions arising from different oncogenic pathways (e.g., HPV-related lesions or chronic dermatosis such as LS) can coexist. In these cases, a proper diagnosis is mandatory to define the correct management.

EMPDV has no pathognomonic symptoms or specific clinical aspects. Lesions often have a pink or red eczematous aspect; reddish erythematous plaque with typical white scaling can be observed and islands of thick white hyperkeratosis can be present. Itching and burning are the most frequent symptoms and, in a few cases, chronic vulvar pain can arise. In several cases, EMPDV can be completely asymptomatic.

Unfortunately, the main clinical signs and symptoms of EMPDV are similar to those observed in women with VIN, LS or other clinical conditions (e.g., vulvar psoriasis, lichen ruber planus, irritative vulvitis, etc.); thus, a differential diagnosis can be extremely difficult because different clinical conditions can coexist in the same woman. However, different vulvar lesions require different management and a different post-treatment follow-up strategy because they have a different risk of progression to cancer.

As shown, the association between EMPDV and vulvar HSIL (formerly known as U-VIN) is the most commonly reported in the literature. When this association occurs, a complete evaluation of the lower genital tract (with a pap smear, HPV test and colposcopy) is advisable because multiple HPV-related lesions can be present, especially in immunocompromised patients [31]. In the case of VIN, a small surgical excision is usually performed because a free margin of a few millimeters is considered to be safe; positive margins do not predict the development of invasive disease and the need to re-excide the tissue around the scar remains to be demonstrated [32]. However, if EMPDV and VIN coexist, a wider surgical excision should be performed because a free surgical margin of at least 1–2 cm is advisable in women with EMPDV [1].

In previously published studies, the association between EMPDV and LS was reported to be extremely uncommon. However, in our series, this association was the most frequently observed (63.6%). The meaning of this correlation is unknown; however, when this contingency occurs, physicians should be careful to not overlook it. Even if LS is not considered to be a proper precancerous lesion due to its intrinsically slow nature, it might prepare the field for a forthcoming SCC [17,18,33]. A long-term follow-up (and a proper corticosteroid therapy) is, therefore, recommended in these cases.

In previous published research, 3 cases of concomitant EMPDV and vulvar SCC/AC were described. In our series, we observed one case of concomitant EMPDV and vulvar cancer (FIGO stage 1B SCC), with a global incidence of 1.5% among all the women included in our analysis. The peculiar clinical appearance of EMPDV (especially in the case of wide eczematous–erythematous lesions with a vulvar, perineal and perianal involvement) can hide a small SCC. In these cases, a proper histopathological diagnosis is mandatory to avoid an undertreatment of the lesion and a radical vulvectomy (rather than a skinning vulvectomy) and lymph-node assessment are necessary in these women [34,35].

Comparing the 22 women with EMPDV and concomitant vulvar premalignant/malignant lesions and the remaining patients with only EMPDV, we found no difference in the mean age. On the other hand, women with EMPDV and concomitant premalignant/malignant vulvar lesions had wider lesions, more frequently involving not only the vulva but also the perineum and perianal region. Interestingly, in women with EMPDV and concomitant vulvar premalignant/malignant lesions, a significantly higher rate of invasive EMPDV was noted. The precise meaning of these findings is unknown and deserves future studies.

As previously highlighted, the vulva is a peculiar anatomical district where several premalignant and malignant lesions arising from different oncogenic pathways can coexist. Therefore, the possibility of not only synchronous but also metachronous lesions should be considered. This is extremely important during the post-treatment follow-up and gynecologists should always be aware of this possibility in order to avoid a potentially harmful misdiagnosis of different vulvar diseases.

The main strength of this study was the large number of cases reported (even considering that EMPDV is an uncommon disease). Moreover, all the cases were diagnosed and treated in referral centers by experienced gynecologists and pathologists.

However, a few potential limitations of this study deserve to be highlighted. Due to its retrospective nature, the clinical and histopathological data available were limited to those already reported in medical charts and a further analysis was not possible. Similarly, a pathology review of all the cases was not performed.

## 5. Conclusions

The association between EMPDV and other premalignant/malignant vulvar lesions is not as uncommon as previously reported, but its specific meaning is still unclear. However, a few questions remain:

- Can the association between EMPDV and VIN, LS or SCC be clinically relevant or determine a different risk of recurrence?
- Is there a real increased risk of invasive EMPDV in women with synchronous VIN or LS? Is there an etiopathogenic explanation for this finding?
- In the case of coexisting lesions, should a different therapeutical approach be used?

Considering the low incidence of EMPDV, it is currently impossible to answer such questions and future studies are advisable. Moreover, the potential overlapping features between different vulvar lesions (even with immunohistochemical staining) highlights the importance of a “centralized” evaluation in referral centers of all women with EMPDV by dedicated gynecologists and pathologists with a particular expertise.

## Figures and Tables

**Table 1:** Cases of EMPDV and concomitant vulvar cancer and precancer.

Case N°	Age at Diagnosis	Type of EMPDV	Site	Number of Lesions	Concomitant LS	Concomitant VIN	Concomitant SCC or AC
1	65	Type 1a	Vulva	Multiple	Yes	No	No
2	71	Type 1a	Vulva	Single	Yes	No	No
3	35	Type 1b	Vulva	Single	No	Vulvar HSIL	No
4	53	Type 1a	Vulva/perianal	Multiple	Yes	d-VIN	No
5	79	Type 1a	Vulva/perineum/perianal	Multiple	Yes	d-VIN	No
6	56	Type 1b	Vulva	Single	Yes	no	No
7	72	Type 1b	Vulva	Single	Yes	d-VIN	No
8	83	Type 1b	Vulva/perineum/perianal	Multiple	No	d-VIN	No
9	65	Type 1a	Vulva	Multiple	No	d-VIN	No
10	65	Type 1a	Vulva	Single	No	d-VIN	No
11	64	Type 1a	Vulva/perineum/perianal	Single	Yes	Vulvar HSIL	No
12	74	Type 1a	Vulva/perineum	Multiple	No	d-VIN	No
13	59	Type 1a	Vulva/perineum	Single	No	d-VIN	No
14	37	Type 1b	Vulva/perineum	Single	No	No	No
15	64	Type 1b	Vulva	Single	Yes	No	No
16	69	Type 1a	Vulva/perineum/perianal	Single	Yes	No	No
17	68	Type 1a	Vulva/perineum	Single	Yes	No	Yes
18	84	Type 1a	Vulva	Single	Yes	No	No
19	53	Type 1a	Vulva	Single	Yes	No	No
20	81	Type 1a	Vulva/perineum/perianal	Single	No	d-VIN	No
21	63	Type 1b	Vulva	Single	Yes	No	No
22	53	Type 1a	Vulva	Multiple	Yes	No	No

Type 1a: intraepithelial EMPDV; type 1b: invasive EMPDV; VIN: vulvar intraepithelial neoplasia; vulvar HSIL: high-grade squamous intraepithelial lesion; formerly known as usual-type VIN and HPV-related; d-VIN: differentiated VIN; LS: lichen sclerosus; SCC: squamocellular cancer of the vulva; AC: vulvar adenocarcinoma.

**Table 2:** Comparison between women with EMPDV and concomitant vulvar premalignant/malignant lesions and women diagnosed only with EMPDV during the study period.

Characteristics	EMPDV + Concomitant Premalignant/Malignant Vulvar Lesions (*n* = 22)	EMPDV (*n* = 47)	*p*-Value
Age, mean (±SD)	64.2 (±13)	66.3 (±11.6)	0.50
Invasive EMPDV, *n* (%)	7 (31.8%)	5 (10.6%)	0.03
Vulvar and perineal (and/or perianal) involvement, *n* (%)	10 (45.5%)	10 (21.3%)	0.04
Multiple lesions	7 (31.8%)	12 (25.5%)	0.59
History of vulvar SCC/AC	-	-	-

EMPDV: extramammary Paget’s disease of the vulva; SCC: squamocellular cancer of the vulva; AC: vulvar adenocarcinoma.

**Table 3:** EMPDV associated with one or more concomitant premalignant and/or malignant vulvar lesions (literature review).

Authors	N° of Cases	Age at Diagnosis	Histological Findings	Primitive Origin	Concomitant Lesions
Hawley et al., 1991 [22]	1	66	EMPDV	Vulva	U-VIN (VIN 3) + invasive AC
Brainard et al., 2000 [23]	2	66	EMPDV EMPDV	Vulva Vulva	U-VIN 3 + SCC
Orlandi et al., 2001 [16]	2	64, 72	EMPDVEMPDV	Vulva Vulva	LS + d-VIN (VIN 2); VIN 3
Matsumoto et al., 2007 [27]	1	61	EMPDV	Vulva	U-VIN (VIN 3)
Kim et al., 2011 [25]	1	47	EMPDV	Vulva	U-VIN (VIN 3)
Goyal et al., 2014 [26]	1	57	EMPDV	Vulva	U-VIN (VIN 3)
Jang et al., 2016 [13]	1	68	EMPDV	Vulva	U-VIN (VIN 3)
Onaiwu et al., 2017 [24]	1	67	EMPDV	Unknown	SCC
Samiee-Rad et al., 2020 [12]	1	59	EMPDV	Vulva	U-VIN (VIN 3)

EMPDV: extramammary Paget’s disease of the vulva; VIN: vulvar intraepithelial neoplasia; U-VIN: usual-type VIN; d-VIN: differentiated VIN; LS: lichen sclerosus; SCC: squamocellular cancer of the vulva; AC: vulvar adenocarcinoma; type 1a: intraepithelial EMPDV; type 1b: invasive EMPDV; type 1 c: EMPDV with an underlying adenocarcinoma of the skin.

## Data Availability

Data used for the present study are available on request from the corresponding author.
